# Self-Efficacy, Job Satisfaction and Teacher Well-Being in the K-12 Educational System

**DOI:** 10.3390/ijerph182312763

**Published:** 2021-12-03

**Authors:** Florica Ortan, Ciprian Simut, Ramona Simut

**Affiliations:** 1 The Teacher Training Department, University of Oradea, 410087 Oradea, Romania; 2 Department of Philosophy, Nelson Mandela University, Port Elizabeth 6031, South Africa; Cipriah.Simut@mandela.ac.za; 3 Department of Economics and Business, University of Oradea, 410087 Oradea, Romania; simut.ramona@yahoo.com

**Keywords:** job satisfaction, self-efficacy, collegiality, working conditions, student behavior, professional development, well-being

## Abstract

Teacher job satisfaction and well-being have a significant impact on educational outcomes, considering that teaching is the main objective of the educational process. The aim of this study is to examine the relationship between teacher job satisfaction and four main categories of determinants: self-efficacy, relational aspects (colleague collaboration, student behavior, school management), work-related aspects (administrative workload, teaching tasks), and working conditions, in order to identify various implications for teachers’ well-being. The study employs a survey delivered to 658 K-12 (pre-university) teachers, from the North-West region of Romania. We used factorial analysis and a structural equation model to test eight proposed hypotheses. The results showed that self-efficacy, promotion, positive student behavior, and working conditions have significant effects on job satisfaction. These factors influence job satisfaction and well-being in the teaching profession because they ensure a positive work environment in which teachers and students thrive, thus leading to higher levels of involvement from teachers, students, and parents alike. An efficient work environment decreases attrition, burnout, emotional exhaustion, and teacher turnover, while increasing job satisfaction, well-being, and teacher retention.

## 1. Introduction

The Romanian educational system is highly controversial, bureaucratic, and unstable. In the last three decades, a series of modifications have been implemented in the system, under the guise of reform, which never seem to achieve their proposed goals. In the last decade, controversy broke out regarding poor student results, visible in their periodic examinations and at the baccalaureate. The issue put even greater pressure on teachers’ qualifications and their efficiency in class, drawing public reproach to a profession which had to deal with constantly limited funding; a situation which does not seem to change, regardless of the political color of the education ministers. Under-financing has disastrous consequences for the entire educational system [1]. One of the recent controversies revolved around the poor results that Romanian students obtained on the PISA tests [2]. The situation maintains constant pressure and media outrage directed at the efficiency of teachers. The most recent educational reform centers on the Educated Romanian program, which has come under serious scrutiny due to its lack of substance and institutional delays. The system is also plagued by frequent curriculum changes that are not in tune with market demands, and a high number of blocked teaching positions result in teachers being unable to advance professionally [1].

There is an important difference that must be noted between the general term “well-being” and the term “occupational well-being”. Well-being can be defined, as McCallum and Price [3] point out, as being diverse and fluid but connected to individual, family, and community beliefs and values. It is also constructed around culture, opportunities, and temporal contexts. In essence, they argue, general well-being is about positive notions, but is unique to every individual, providing a sense of identity and demanding respect. The definition given by van Horn et al. [4] is that occupational well-being is “the positive evaluation of various aspects of one’s job, including affective, motivational, behavioral, cognitive, and psychosomatic dimensions”. Occupational well-being is connected to work engagement and career choice satisfaction, as Matteucci et al. [5] observe, and it is strongly dependent on emotional exhaustion and job satisfaction. On a more specific note, Acton and Glasgow [6] defined teacher well-being as “an individual sense of personal professional fulfilment, satisfaction, purposefulness and happiness, constructed in a collaborative process with colleagues and students”. This article will use the definitions of occupational and teacher well-being, written simply as “well-being”, and not as “occupational well-being” or “teacher well-being”.

Zoller and Bacskai analyze fundamental aspects of job satisfaction in the Romanian educational system, namely, job satisfaction, self-efficacy, and school climate, concluding that job satisfaction, in Romanian lower secondary education, is dependent on professional development, a disciplined atmosphere, teacher–student relationships, and stakeholder relations [7]. Despite various issues, teachers’ job satisfaction and well-being depend on relationships; therefore, policy makers might consider the impact of their implemented policies on school climates. In this context, teacher job satisfaction and well-being are aspects that need to be studied, to generate genuine change in the educational system [8]. Furthermore, despite the systemic issues, both job satisfaction and well-being are strongly related to school management, which can create the proper working environment for the two elements to improve. Besides the management aspect of the issue, teachers deal with two more fundamental elements: relations with students, colleagues, and parents, and professional advancement [9,10].

In the pre-university system, job satisfaction is strongly related to job experience and professional development. It seems that younger teachers, who are at the beginning of their professional career, have higher levels of job satisfaction than older, more experienced teachers. However, the younger teachers are also more prone to leave the profession, compared to the older teachers. On the same note, job security, through tenure [11], provides increased job satisfaction to older, more experienced teachers, whereas younger teachers who do not have tenure show lower levels of job satisfaction [11,12,13,14,15,16]. Despite the negative aspects, the teaching profession still holds prestige, and personal and social value [17]. The symbolic capital of the teaching profession increases social status, which leads to an increase in support for certain schools where power of decision, work style, personal independence, intelligence level, and teaching abilities are visibly promoted [18,19,20,21,22,23].

The tipping point of the balance between teacher job satisfaction, well-being, and the school environment, is related to how efficient school management is in reducing bureaucracy and allowing teachers to focus on teaching and building meaningful relationships with other colleagues and with students, rather than having to carry unnecessary additional workload [19].

The present study aims to explore and analyze the factors that influence job satisfaction among K-12 teachers, based on the identified relationships between the factors and job satisfaction, in order to offer solutions and proposals to improve well-being for Romanian teachers. To achieve this goal, a questionnaire survey was used for the collection of statistical data and a factorial analysis was conducted to compute the indexes for our study. This paper is organized as follows: (1) Introduction; (1.1.) Literature Review (with an overview of specialized literature regarding the job satisfaction and well-being, and a presentation of the purpose and the objectives of the study); (1.2.) Research Hypotheses (with a description of hypothetical relations); (2) Materials and Methods (with a research method, a respondent’s profile and the conceptual model); (3) Results; (4) Discussion and Practical Implications; (5) Conclusions.

### 1.1. Literature Review

The specialized literature on job satisfaction reveals that it is an integral part of any industry or work environment. In the case of health workers, it was found that ethical leadership positively and significantly influences job satisfaction, by: applying ethical principles to problem-solving and equity [24]; offering training and organizational support [25] together with management support and resources, despite tendencies to quit the job [26]; offering psychological support despite difficult working conditions [27]; prompting risk prevention plans [28]; offering improved health maintenance programs [29]. In all the previously mentioned sources, well-being was either positively or negatively affected by management decisions. In the case of Japan’s civil servants’ sector, job satisfaction was influenced by satisfaction with job interest, skills, and how abilities were used, while satisfaction with how a section was run, co-workers, work prospects, physical working conditions, and payment had a lower influence [30]. For workers in other sectors of industry that are considered hazardous, such as Turkish jean sandblasting workers, dock workers, factory workers, and miners, lack of skills lead to low wages and almost no insurance or health care, and hence a high risk of stress and low job satisfaction levels. Sandblasting and dock workers reported the lowest satisfaction level. Despite the hazardous nature of their jobs, miners showed higher levels of job satisfaction than other workers [31]. In the case of Filipina migrant domestic workers in Hong Kong, migration itself was a source of high stress. In this case, salary was the most important determinant of job satisfaction, since this was the main reason for migrating, overtaking the usual factors that are important for job satisfaction such as educational level, previous occupations, and even personal aspirations [32]. Carvajal et al. [33] investigated how gender and age variations impacted job satisfaction in the case of pharmacists in the US. They found that female pharmacists were consistently more satisfied with their jobs than their male counterparts when age was controlled for. During our research, it was observed that job satisfaction is studied mainly among high-profile jobs such as health workers, and to a lesser degree in other industries. Considering other industries, we aimed at presenting a general perspective that would show similarities and differences between various types of jobs and teaching.

Job satisfaction has received great attention in the past century [34]. The most cited definition was given by Locke [35], who defined it in terms of the positive appreciation of one’s own job, which generates a positive emotional state, meaning that job values are fulfilled. In a later article, Henne and Locke [36] elaborated on the nature of job satisfaction, presenting it in relation to the work itself, which needs to be personally interesting and significant, successful, and able to generate accomplishment, progress, growth, responsibility, autonomy, role clarity, role congruence, (lack of role conflict), feedback about performance, and a lack of physical strain. As Ho and Au suggested, in teaching, job satisfaction is the relation between what a teacher wants from the profession and what the teacher perceives it as giving or entailing, resulting in a product that stems from attitudinal and affective responses [37]. On the issue of the development and analysis of job satisfaction, Zhu offers an encompassing perspective [38].

Toropova et al. [39] make a series of important claims arguing that, in the case of job satisfaction, there have been important findings that show a significant association between job satisfaction and the factors studied in this article, such as working conditions, collegiality, workload, and student behavior. Gender is also related to job satisfaction, as women teachers have more self-efficacy, engage more in professional development, and thus have higher levels of job satisfaction, whereas men tend to focus more on collegiality/cooperation. On the same note, teachers with lower self-efficacy tend to have more issues with student discipline and behavior. The wider context shows that schools where there is higher leadership/management support, where student discipline is respected, and where teachers have higher levels of decision-making and autonomy tend to have better teacher retention [39]. From the same perspective, Plopeanu et al. [40] suggest that job satisfaction is made up of personality characteristics and behavior, intrinsic and extrinsic values, work situation, life satisfaction, and social influence, which makes it increasingly difficult to study because teachers interact with one another, adapt to various working conditions, and develop in their careers through development programs. In this context, it is important to draw a distinction between two dimensions of factors that make up job satisfaction, namely, extrinsic and intrinsic dimensions. The intrinsic factors of job satisfaction refer to achievement, recognition, responsibility, advancement, growth, and the work itself. The extrinsic factors refer to supervision, working conditions, co-workers, pay policies, procedures, status, and personal life. In this research, we considered both extrinsic and intrinsic factors, therefore retaining the general formulation of “job satisfaction”.

Well-being is notoriously difficult to define and there is no consensus on what the construct of the concept should be. Despite the difficulties, it has been suggested that it could be split into objective well-being (measurable elements: economic resources, political circumstances, physical health conditions, number of social relationships, literacy) and subjective well-being (subjective experiences: happiness, emotions, engagement, purpose, life satisfaction, quality of social relationships, competence, accomplishment) [41]. As health is not merely the absence of disease or infirmity, well-being is part of health [42]. In the field of education, subjective well-being is considered most frequently [43]. Considering the fact that the present research was performed in a European context, where well-being revolves around individual achievement and self-esteem [44], the definition of Acton and Glasgow [6] was proposed, which describes well-being as an “individual sense of personal professional fulfillment, satisfaction, purposefulness and happiness, constructed in a collaborative process with colleagues and students”. In essence, there is no fixed definition of well-being; instead, it must be considered to be dependent on relationships, specific situations, productivity, and the ability and willingness to engage in various life experiences [45]. Considering the definitions provided for both teacher and professional well-being and job satisfaction applied to teaching, the former includes the latter, meaning that well-being revolves around cognitive and emotional factors more than job satisfaction. Elements such as happiness and purposefulness make well-being different from job satisfaction, which is more pragmatic, revolving around the direct exchange between one’s skills and their appreciation, and the results within the workplace. The research aims at analyzing the factors that contribute to job satisfaction in the K-12 Romanian system and providing a means to identify the impact on teacher well-being.

In other occupations such as fishing, where gender is an issue to be considered together with household care [46], mining, where there is no impact on family life but a significant impact on the environment, which in turn may affect well-being [47], and casinos, where job satisfaction is low due to inherent job requirements such as unsocial hours and limited promotion, issues cannot be addressed by management since they are inherent to the industry [48]. In other situations, such as among migrants, depending on the context, job satisfaction and well-being depend on factors such as cultural background, religiosity, and working conditions, especially when work-related abuse is discovered [49]. For self-employed workers, [50] and manufacturers, who focus more on health [51], job satisfaction is influenced by motivation, performance, job retention, working environment, on-job behavior, and most of all on time management decisions and policies. Well-being is a result of these various factors, which when favorable, tend to offer employee satisfaction, self-worth, and a sense of belonging. Well-being, objective or subjective, is difficult to define, but it considers evaluative or cognitive (general life satisfaction), eudemonic (life purpose), and emotional (happiness, joy, sadness, worry) dimensions, becoming more important at a national level, since it benefits society [52] at a local, as well as a global level [53].

Job satisfaction is also studied in the field of education. Several research papers focus on the determinants of job satisfaction, such as school organization climate [39,54], self-efficacy [39,55], teacher mobility [9,56], workload, commitment, morale [57], and participation in decision-making [58]. The objective of this study was to identity four main determinants, with several sub-determinants, as follows: teacher self-efficacy, working conditions (workload, daily tasks), teacher relations and collaborations (collegiality, student behavior, support from management, educational resources), and professional development/promotion. These determinants were identified in several schools from the North-West region of Romania through an online questionnaire, answered by 658 teachers from the K-12 (pre-university) state-funded educational system.

Teacher well-being is highly dependent on factors that produce attrition, burnout, and teacher turnover. It was found that teachers deal with significantly high levels of stress and low levels of well-being [59]. Other studies show that teachers may have high levels of stress but they also have high levels of job satisfaction, as Kyriacou highlights [60]. One study pinpointed work overload as often the highest stress trigger, as Austin et al. showed [61]. In this context, the work of Pithers warns against generalizations about stress-related issues among teachers that may not have been tested for validity and reliability [62]. Teacher self-confidence, the sense of personal agency and resilience [63], plays an important part in developing well-being in the school environment by reducing stress and attrition through development and well-being programs designed for the teaching profession [64].

### 1.2. Research Hypotheses

This section presents a theoretical perspective on job satisfaction and the determinant factors that influence it in the working environment. Starting from the literature review, we aimed at identifying the most important factors that influence job satisfaction. Following on from the results, we proposed a series of hypotheses that would aid us in analyzing the relationship between job satisfaction and these determinants, among teachers from the K-12 educational system in the North-West region of Romania.

#### 1.2.1. Self-Efficacy

The term “self-efficacy” was defined by Bandura in 1977 [65], and it can be applied to education in terms of the conviction of teachers that they can execute behavior that produces results or outcomes. As Roberts et al. concluded, teacher self-efficacy increases through in-service programs, even if teachers begin with low self-efficacy [66]. For teachers in primary schools, the results are in tune with current literature that highlights the difficulties faced by teachers in developing their self-efficacy. Although self-efficacy begins with a teacher’s own experiences, and even if it develops as experience is gained, personal beliefs about self-efficacy tend to become rigid. Self-efficacy should focus on what a teacher can do, especially in a particular subject, not as an overall perspective [67]. Sandholtz and Ringstaff obtained positive results for teachers who worked in challenging environments, after a three-year professional development program [68]. Coupled with practice and identity, self-efficacy has positive results for teacher retention, as Polizzi et al. showed, because teacher communities of practice are organized as networks [39,55,69,70]. Velthuis, Fisser, and Pieters found that pre-service improvement programs increased teacher self-efficacy, meaning that universities should improve their educational offer [71]. Wang and Tsai argued that science-teacher hardiness, which is a result of teacher self-efficacy, prompts science hardiness in students, aiding them in developing self-efficacy, but it must be coupled with teacher support [72]. As Dalioglu and Adiguzel [73] found, teacher self-efficacy does not change, under certain conditions, with regard to class management, but it does improve student teaching levels. Whether face to face or online, teacher job satisfaction is also related to factors such as teaching flexibility regarding when, where, and how teaching occurs, because this allows teachers to meet student needs, gives more time for interacting individually with students, and, finally, offers teachers satisfaction when the efforts they put into teaching have results in student performance, as Borup and Stevens found [74].

Self-efficacy is not the same in all countries, as Avalos and Bascope showed, proving that without adequate implementation and expert support, teachers’ preparation will be lacking [75]. Lamote and Engels found that self-efficacy decreased when student teachers engaged in classroom practice following a pupil-centered approach, where focus on the subject matter, classroom order, and long-term qualifications decreased. First-year students tended to have content-oriented views of learning and teaching, but these developed in the second and third years into pupil-oriented views [76]. As years of experience accumulated, self-efficacy increased, but after about 23 years it began to decline, as Klassen and Chiu observed [67]. Teachers’ self-efficacy can be expanded and perfected through specialized training programs that have a high level of complexity and require teachers to delve deeper into their knowledge of teaching and learning, as shown by Catalano, Albulescu, and Stan [77]. Teacher self-efficacy is improved by teacher interaction, which positively affects student self-efficacy, as proven by Hwang and Ham [78]. Zakariya [55] provided an ample study that proved the connection between teacher self-efficacy, job satisfaction, and school climate. By utilizing structural equation modelling, he concluded that between school climate and job satisfaction there is a strong direct relationship. In addition, he identified a strong and direct relationship between self-efficacy and job satisfaction. All the three self-efficacy types that he studied had an impact on job satisfaction, but the greatest influence was exerted by self-efficacy in instruction. Studies by Edinger and Edinger [79] and Skaalvik and Skaalvik [80] arrived at similar results, even though the dimensions of teacher self-efficacy were slightly different. Molero et al. [81] used cluster analysis for 500 high school teachers and found that low levels of burnout seemed to increase self-efficacy, which in turn increased job satisfaction. A study by Capone and Petrillo [82] argued that fewer teachers claimed to be flourishing in their job, with a lower prevalence of depression and burnout but higher levels of job satisfaction and self-efficacy, while most were moderately satisfied. In the context of self-efficacy, mastery of a certain subject was not enough to prepare future generations of students. Teachers are required to develop self-efficacy also in relation to personal and social skills, and also to develop active, involved, and engaging teaching–learning experiences that are combined with well-being [83]. In a similar manner, another study, by Yang et al. [84], argued that in Sweden, based on the TALIS 2013 document, the most substantial total effect on job satisfaction, came from self-efficacy, with a direct effect of 0.15 and an indirect effect of 0.07. The same significant effect was reported for Norway also, with the highest total effect of 0.28. The results are not the same in all studies. Shaukat et al. [85] found that there was no significant correlation between a teacher’s self-efficacy, beliefs, and job satisfaction, but a key factor was the school environment where teachers worked with children with disabilities.

**Hypothesis** **1** **(H1).** *Teachers’ self-efficacy has a positive effect on teachers’ job satisfaction. Teachers’ self-efficacy is the strongest predictor of job satisfaction.*

#### 1.2.2. Working Conditions

Teaching relies on creativity, which takes up time. Selamat et al. [86] have showed that teachers’ job satisfaction diminishes when the workload increases, arguing that organizational climate is important for job satisfaction. Organizational climate proved to be an important factor that had a high influence on job performance, and it rested on the support and hindrance resulting from leadership and teacher behavior. Ghavifekr and Pillai [54] considered that organizational climate increased job satisfaction and teacher retention. The results also showed that work overload could hamper teaching as a main educational objective, even if working conditions had a relatively low impact on teacher job satisfaction compared to other analyzed factors. Knox [87] pointed out that routine tasks lower job satisfaction, whereas flexibility and a sense of the importance of teaching, coupled with the conviction that teachers can make a difference in the lives of students, results in greater job satisfaction. Kloep and Tarifa [88] argued that despite economic and physical conditions, at least to a certain extent, teacher job satisfaction and class involvement can be high, provided professional autonomy and social support exist [88,89]. Furthermore, Kloep and Tarifa concluded that, in the case of Albania, teachers considered material rewards to be highly valued, but their overall significance for job satisfaction was lower than job autonomy, described in terms of opportunities and challenges. As Abu Taleb [90] pointed out, teachers may have average job satisfaction levels, but this depends on working conditions coupled with children’s behavior and parents’ involvement. Job satisfaction among kindergarten teachers is ranked as average. In a wider context, teacher job satisfaction influences children’s educational outcomes. The issue of excessive workload has been identified as leading to emotional exhaustion, teacher turnover, and attrition among teachers [91]. Job satisfaction is influenced by the workload, which translates into hectic schedules and many interruptions from tasks that are already in progress, amounting to too many tasks in too little time. Rest and relaxation were also affected. Teachers expect to have a high workload in their profession, but if it becomes highly bureaucratic and takes up too much time from other teaching activities, it contributes to lower well-being, even if the excuse for engaging in such tasks is related to the success of the entire department or the entire institution. Raza and Arid [92] showed that teacher job satisfaction decreased when teachers had to deal with clerical tasks unrelated to teaching. However, teachers considered preparing class notes, keeping class attendance records, and recording test marks as clerical work that, albeit necessary, still affected job performance.

Teacher daily activity is not reduced only to the act of teaching, but it encompasses administrative and teaching tasks that can affect work intensity. Ballet and Kelchtermans argue [93] that this is considered challenging and complex [94], generating decreased job satisfaction [95]. Tasks are still considered to be what defines part of a teacher’s self-identity, together with self-image, self-esteem, job motivation, and future-oriented perceptions [96]. Canrinus et al. [97] argued that job motivation, self-efficacy, occupational commitment, and changes in the level of motivation lead to a sense of professional identity in which tasks are seen as an important part of strong work satisfaction and work engagement, as also found by Li, Liu, and Zhang [98]. It seems that teachers with high levels of self-efficacy have more confidence in engaging with and completing school-related tasks, which leads to increased job satisfaction, as shown by Peng and Mao [99]. Anastasiou and Belios [100] conducted research aimed at identifying how age impacts job satisfaction and emotional exhaustion in primary school teachers in Greece. The age range was from 20 to beyond 50, while work experience ranged from 0 to over 21 years, and the study included both female and male teachers. The results showed that emotional exhaustion was negatively influenced by job satisfaction and age, caused most frequently by extrinsic job characteristics such as working conditions and working hours. Liu and Ramsey [101] analyzed the situation in the United States and found that teachers were least satisfied with regard to compensation and work conditions. They considered that work conditions did not allow them enough time to prepare and plan classes. Workload was considered excessive during a typical school week.

**Hypothesis** **2** **(H2).** *Workload has a negative effect on job satisfaction.*

**Hypothesis** **3** **(H3).** *Daily tasks have a negative effect on job satisfaction.*

#### 1.2.3. Teacher Relations and Collaborations

Hewett and La Paro [102] defined collegiality as social interactions and a feeling of belonging within the teacher community, which contributes to program quality but not classroom quality. As Hur, Jeon, and Buettner [103] pointed out, collegiality contributes to increased job satisfaction and leads to positive child-centered beliefs. The perceived collegiality and teacher influence was positively associated with teacher job satisfaction, and in turn with child-centered beliefs. In other contexts, reforms can have negative effects, such as compromised teacher collegiality, which lead to lower job satisfaction, higher levels of stress, and teacher turnover, as pointed out by Liu, Xu, and Stronge [104]. Mieke and Vandenberghe [105] assert that both autonomy and collegiality need to be considered for increased job satisfaction and positive working conditions. Autonomy and collegiality need to be assessed in a balanced way, because they have certain forms, which coupled with workplace conditions seem to have a higher positive influence on the professional development of teachers. Collaboration is part of the teaching profession; therefore, efficient collaboration with colleagues [7] increases well-being and has beneficial effects on the classroom environment, as Yang et al. [84] pointed out. Zoller et al. [7] observed that teachers have an internal desire to learn and develop professionally. In the case of Romanian teachers, effective professional development programs have a high impact on job satisfaction. The variables of the methodological culture dimension, such as teacher effectiveness, disciplined atmosphere, and teacher–student relationships, also influence job satisfaction.

Teachers are involved to a high degree in education-based student relationships, having a basic need for relatedness, as Spilt, Koomen, and Thijs argued [106]. Further, Lavy and Bocker presented the case for teachers’ sense of meaning and sense of self affecting teacher–student relationships, as well as job satisfaction [107]. Gender seems to be an important factor in how the school environment and student relationships are perceived and evaluated, according to the study by Huang [108]. Furthermore, it appears that student–teacher relationships are important for increasing job satisfaction in veteran teachers, as pointed out by Admiraal et al., who also showed that veteran teachers who have lower job satisfaction can be helped through sustained support [109]. In the case of disruptive students, collaboration between the teacher and principal seems to be efficient in solving classroom issues and increasing teacher job commitment by offering a sense of belonging, according to Collie [110]. Gil-Flores [111] found that positive teacher–student relationships, where the teacher listens to the students and therefore promotes the student’s well-being, played the most significant role in the prediction of teacher job satisfaction. Madero [112] contributed to the debate, claiming that, in agreement with the literature, less-dissatisfied teachers collaborated efficiently with their peers and also operated in a culture of school participation. His study refers to secondary teachers in schools from Mexico, Chile, and Brazil. Along the same lines, Zakariya [55] argued that job satisfaction is influenced by a direct positive effect of teacher–student relations. Buonomo et al. [113] conducted similar research on job satisfaction, analyzing whether collective beliefs and emotions regarding the professional role were a predictor of job satisfaction. The age range varied from 26 to 65, and the job experience ranged from 1 to 41 years. Their findings showed that job satisfaction increased when the teaching profession was considered in terms of being part of a professional community, with positive relations with colleagues, students, and families.

Teachers who are professional and understand their jobs have increased well-being, provided they are allowed to do what they know is worth doing, as a result of strong beliefs and values, which increase student well-being and hence the quality of teaching programs, as Hall Kenyon et al. [114] found.

Hewett and La Paro [102] defined supervisor support as teachers’ perceptions of leadership and the support offered. In this sense, Da’as [58] made the case for principals using cognitive skills to limit absenteeism, rather than exceptional or charismatic behavior, thus increasing organizational performance. This finding led to a similar one, posited by Ghavifekr and Pillai [54], who argued that within a well-organized school climate, the responsibility factor increased job satisfaction. Cann et al. [115] explained how leadership positively influences job satisfaction and enhances well-being, defined as feeling valued, having meaningful professional development, and having a part in decision-making. Chong, Mansur, and Ho [116] encouraged leadership development for principals, as it increases job satisfaction and teacher retention. The extent of professional involvement of teachers in the decision-making process has an important outcome in the development of school policies [117]. The study conducted by Yao, You, and Zhu [118] showed that if teachers receive adequate support from management, their affective commitment increases and is followed by positive job performance, hence generating increased job satisfaction. In addition, in another quantitative study, Sun and Xia [119] analyzed the relationship between the perceptions of the teachers of school-climate leadership, job satisfaction, and teacher self-efficacy, using multi-level structural equation modelling. The results showed that leaders who include teachers in the leadership decision-making process, have a positive effect on job satisfaction. Ainley and Carstens [120] found that schools that have interactions between school principals, teachers, and students, in a framework of distributed leadership, are more likely to have teachers with higher job satisfaction levels. The findings are similar to those of Torres [121], Sims [122], and Liu and Werblow [123], who found a positive relationship between effective leadership and job satisfaction. Liu and Werblow [123] also suggested that an important factor in increasing job satisfaction among principals and teachers, at both personal and organizational levels, was strongly dependent on the manner in which principals and teachers develop collegiality and team leadership in instructional management.

Teacher well-being in the educational system refers to feeling valued, meaningful professional development, and being involved in decision-making. Well-being is achieved mainly when decision-makers, such as principals or school managers, show skills such as relationship building, contextual competence, and social and emotional competence [115]. The leadership style can have positive effects on job satisfaction by enhancing workforce performance and organizational goals, through motivation and a gradual increase of organizational commitment, as Altaf et al. [124] demonstrated. Badulescu, Bungau, and Badulescu [125] argued in favor of learning processes that are aligned with sustainable development, for benefiting and optimizing the educational environment in the long run, after students have graduated.

The teaching environment requires several types of resources, which influence both job satisfaction and well-being, together with teacher engagement, but further research is needed, as Skaalvik and Skaalvik argued [126]. Simbula et al. [127] found that job resources, self-efficacy, and work engagement are related, but over time, leading to the conclusion that teacher engagement depends on both well-resourced environments and self-efficacy. On the same note, Demerouti et al. [128] showed that if job demands are related to teacher exhaustion and burnout, lack of job resources is related to teacher disengagement. Educational resources need to be viewed at a larger scale, to include technological devices, as posited by Lee and Quek [129]. The issue of resources can be differentiated by job resources (such as perceived autonomy support, opportunities for professional learning and relationships with colleagues) and personal resources (such as adaptability, cognitive and behavioral coping, and self-efficacy, which applies to both personal and organization levels) [130,131]. As Kiss [132] argued, the educational system seems to be in a permanent state of transition; therefore, a high degree of flexibility is needed to properly adapt to various new scenarios, that must benefit both teachers and students. Toropova et al. [39] argued that in comparison with other studied factors such as student discipline, teacher cooperation, and teacher workload, the factor of referring to school materials has a lower effect on job satisfaction. Working in a school environment may lead to high strain regarding job demands and resources, because job demands such as workload, disciplinary issues, and time pressure can impact job resources such as perceived autonomy, professional learning, and collegiality, leading to low engagement, burnout, and negative school environment outcomes that will also negatively impact well-being [5,130].

**Hypothesis** **4** **(H4).** *Collegiate cooperation has a positive effect on job satisfaction.*

**Hypothesis** **5** **(H5).** *Respectful behavior of students has a positive effect on teachers’ job satisfaction.*

**Hypothesis** **6** **(H6).** *Support from school management has a significant and positive effect on teachers’ job satisfaction.*

**Hypothesis** **7** **(H7).** *The resources of the educational institution have a positive effect on job satisfaction.*

#### 1.2.4. Professional Development/Promotion

An integral part of the educational system is the possibility of, and the ability to, develop professionally. Despite the possibility existing, there are situations in which teachers can reach a career plateau, which negatively impacts job satisfaction, as Drucker-Godard et al. found [133]. Job satisfaction dimensions may include career development, school management, teacher and research services, salary, and also the work itself, according to Du, Lai, and Lo [134]. An important element of professional development is the freedom to choose such development from the perspective of career advancement [135]. In a broader category, teachers, as knowledge workers, define professional development opportunities as a flexible work schedule and colleague support, as well as work–family relations and job security, which also influence job satisfaction, as argued by Viñas-Bardolet, Torrent-Sellens, and Guillen-Royo [136]. Eren suggested that policy makers need to take into consideration the factors that influence pre-service teachers’ engagement and retention, along with aspirations in the teaching profession and professional development, since these factors may lead to decisions to remain in or leave the profession [137]. Teacher well-being is enhanced through ongoing support to perfect teaching skills and abilities, which are manifest throughout the teacher’s career and which need to be designed and developed, since they will inevitably affect the well-being of students [138,139]. Yang et al. [84], based on an analysis of four countries: Denmark, Finland, Sweden, and Norway, concluded that in Sweden and Norway, the effect of professional development on teacher job satisfaction is significant and direct, while in Denmark and Finland the effect of professional development on job satisfaction is lower. Toropova et al. [39] also concluded that professional development has a positive effect on job satisfaction. The study showed that teachers with longer exposure to their career and to professional development seemed to have higher job satisfaction levels. Along the same line of argument, Sims [140], based on an international perspective, found a positive relationship between professional development and job satisfaction. Ma and MacMillan [141] and Liu and Ramsey [101] reached the same conclusion, but based on an analysis performed in a single country (Canada).

**Hypothesis** **8** **(H8).** *Career promotion has a significant effect on teachers’ job satisfaction.*

## 2. Materials and Methods

### 2.1. Data Collection Procedure and the Sample

The cross-sectional quantitative research was conducted with non-random convenience sampling in order to determine the main factors that influence teachers’ job satisfaction, in order to determine the implications that these determinants have for sustainable education. The proposed model contains nine constructs, namely: job satisfaction (JS), self-efficacy (EFFIC), students’ behavior (STUD), leadership condition (COND), resources (RESO), colleagues’ cooperation (COLEG), career promotion (PROM), workload (WORK), and tasks (TASK). The factors were determined using a questionnaire. The questionnaire was designed to capture as well as possible the specific characteristics of teachers’ satisfaction at work, working conditions, and the relationships they have with students/colleagues. Therefore, the questionnaire was composed of two main parts: the first part contained information about the demographic of the respondents, i.e., gender, age, education level, experience, teaching level, teaching degree, profession type, residence, teaching location, and income level, while the second part contained questions or items that characterized the job satisfaction among teachers (JS) and a series of questions or items that allowed the teacher’s activities at work (relationship with colleagues, relationship with students, relationship with school management, etc.) to be analyzed. In the second part of the questionnaire, all the measurement scales were evaluated on a 5-point Likert scale, where “1 = strongly disagree” and “5 = strongly agree” or “1 = never” and “5 = many times”. Moreover, in order to maintain the uniqueness of the measurements, the questionnaire was written in Romanian and then translated into English. Before applying this questionnaire, we conducted a pilot test on a sample of 40 teachers (10 teachers, 10 middle school teachers, 10 educators and 10 high school teachers) to verify the accuracy and precision of the questions, after which the questionnaire was revised according to the observations received from the 40 teachers.

Therefore, for data collection, the questionnaire was transposed to an online format in Google Forms and sent to teachers from Romania, in Bihor and Satu Mare counties, in May 2021, by email. The data were downloaded from Google Forms into MS Excel, IBM SPSS Statistics 26 (version 26.0.0, New York, NY, USA), and IBM SPSS Amos 26 (version 26.0.0, Amos Development Corporation, Wexford, PA, USA) and verified for coding accuracy. Given that in Google Forms we had the option to make the answers mandatory, the database was complete and did not contain missing data. Descriptive statistical analyses were performed in IBM SPSS Statistics. IBM SPSS Amos was used to test the hypotheses and the model by modelling the structural equation (SEM). Regarding the sample size, Schumacker and Lomax [142] suggested that a minimum of 10–20 subjects per parameter estimated in the model are optimal, while Kline [143] and Hair et al. [144] suggested that there should be a minimum of 10 cases per parameter or item required for the statistical analysis. Regarding the sample size in the situation where we want to determine whether an SEM model is adequate or not, according to Kline [145] and Marsh et al. [146], a sample size of 200 is a suitable minimum for SEM in SPSS Amos. Therefore, a minimum of 420 responses was required, given that the number of items in the proposed model was 42. Therefore, the sample size of 658 respondents exceeded the above and the analysis was justified. Furthermore, for testing SEM measurement models, it is necessary to simultaneously meet a set of conditions [147,148]: the data should be normally distributed, for each latent variable it is recommended to have at least three indicators, to avoid missing data, recursion of relationships, and interval scales, and a reasonable sample size is required relative to the number of indicators in the model, as mentioned above. In the following, we will briefly refer to each step that must be performed in order to build a model using structural equation modelling.

### 2.2. Respondent’s Profile

Among the 658 respondents (see Table 1), 89.75% of the teachers were female and 10.3% were male. Most of the respondents were aged 40–50 (41%), followed by the 30–40 group (25.4%) and the 50–60 age group (20.7%); 3.6% were aged 60 or above and 9.3% were aged 20–30. Moreover, the majority of the respondents had a bachelor’s degree (48.9%), followed by those with a master’s degree (47.3%), and a PhD degree (3.8%). Regarding the teacher’s experience, most of them had over 20 years of experience, 31.6% had between 10 and 20 years, 13.5% between 3 and 9 years and 5.9% had under 3 years. Among the respondents, 4.4% taught in preschool, 43.3% taught in primary school, 26.4% in lower secondary and 25.8% in higher secondary. Most of the teachers had a first degree (68,1%), followed by 19.3% without a degree, and 12.6% with a second degree. Out of the 658 teachers, 415 were teachers, 201 were primary school teachers, 16 were educators and 26 were auxiliary teaching staff.

Another aspect that we analyzed referred to the environment of origin of teachers and the environment in which they taught (urban or rural). We noted that most of those who completed the questionnaire came from urban areas (74%), and only 69% taught in urban areas. The respondents also indicated their income. Most of the teachers had an income of over RON 3000 (77.5%), 17% had an income between RON 2500 and 3000, and approximately 5% had an income between RON 2000 and 2500, while only 5 teachers had an income under RON 2500.

### 2.3. The Model

Well-being, coupled with life satisfaction and quality of life, is connected to the issue of job satisfaction, since this is a significant part of employees’ lives but they are influenced also by unemployment. According to the literature review, there are several factors that influence well-being, such as health and safety or the benefits of a contract, but also job status. Thus, in order to identify the impact that factors such as working conditions, promotion, relationships with colleagues and school management, relationships with students, and daily tasks have on job satisfaction among teachers, we proposed a series of items after studying the literature. Table A1 presents the items with which we studied job satisfaction, working conditions, relationships with colleagues, daily tasks, and self-efficacy, these being adapted largely after Klassen and Chiu [67], Toropova, Myrberg, and Johansson [39], Önder, Akçıl, and Cemaloğlu [56], Szromek and Wolniak [70], and Stevens [149]. Each latent variable was modified by the authors.

The analysis process was started by testing the measurement model through exploratory factor analysis (EFA), starting from the items presented in Table A1. Variables with factor loadings under 0.4 were deleted [150]. Taking into account this condition we had: job satisfaction (JS) measured by five variables (JS1, JS2, JS3, JS4, JS5), the career promotion latent variable made up of three variables (PROM1, PROM2, PROM3), students’ behavior made up of four variables (STUD1, STUD2, STUD3, STUD4), leadership support measured by eight variables (COND1, COND2, COND3, COND4, COND5, COND6, COND7, COND8), resources made up of three variables (RESO1, RESO2, RESO3), relationships with colleagues measured by six variables (COLEG1, COLEG2, COLEG3, COLEG4, COLEG5, COLEG6), workload made up of three variables (WORK1, WORK2, WORK3), self-efficacy measured by seven variables (EFFIC1, EFFIC2, EFFIC3, EFFIC4, EFFIC5, EFFIC6, EFFIC7) and tasks measured by four variables (TASK1, TASK2, TASK3, TASK4). Thus, starting from these indicators, the study aimed at testing the hypotheses, in order to determine the relationship between K-12 teacher job satisfaction and working conditions, relations with students, colleagues and the school management, and the resources that the teachers have at the workplace.

**Figure 1:**
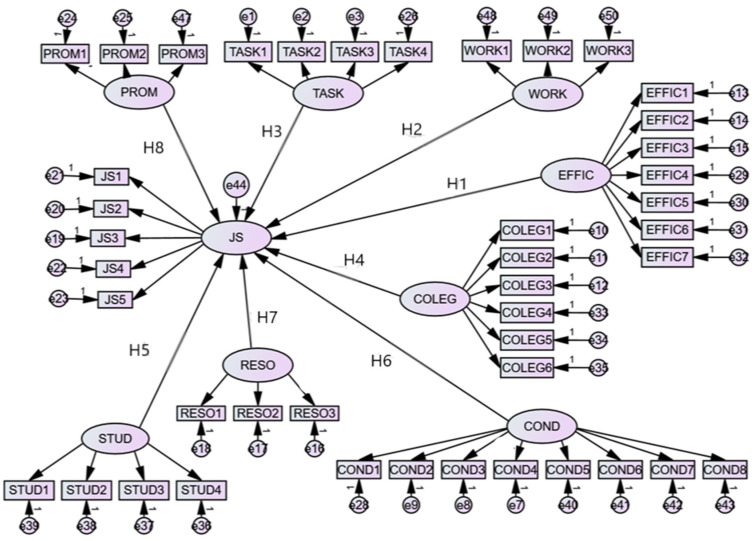
Proposed research model for the study.

The arrows that link latent variables such as JS, PROM, TASK, WORK, EFFIC, COLEG, COND, RESO, and STUD, represent causal relationships in the direction of the arrows (Figure 1). The objectives of this study were to test the eight hypotheses. Error terms for all observed indicators are indicated by e1 to e50.

### 2.4. Data Analysis

In order to analyze the statistical data, we used the statistical software IBM SPSS v. 26.0 (New York, NY, USA) and Amos 26.0 (Amos Development Corporation, Wexford, PA, USA) To test the eight hypotheses stated we used EFA (exploratory factor analysis), CFA (confirmatory factor analysis), and SEM (structural equation modelling). Before performing the EFA analysis, we tested the levels of correlation between the analyzed items. We applied the Kaiser–Meyer–Olkin (KMO) measure of sampling adequacy and the Bartlett test to determine whether there was a sufficiently high correlation to perform the analysis. According to the theory, values of KMO statistics less than 0.50 indicate that the EFA analysis may not be adequate [151,152,153], regarding the sphericity test of Bartlett’s test of the null hypothesis that the correlation matrix is an identity matrix; that is, the variables were indeed uncorrelated. If the statistic *p* was less than 0.10, the null hypothesis could not be rejected, so we could say that the variables were indeed correlated [154]. After checking the level of correlation between the items, we applied the EFA analysis to extract the factors using a varimax rotation. In the analysis, we kept only those items with factorial loads greater than 0.40, while those with loads less than 0.40 were deleted. The next step was the confirmatory factor analysis (CFA), which was performed to first test the overall suitability of the measurement model and then to assess the reliability and validity of the latent variables. The last stage of the study consisted of analyzing the causal relationships of the constructs using structural equations modelling (SEM) and the model fit indexes.

### 2.5. Exploratory Factor Analysis

The first step was to perform the EFA with the 42 items. The KMO statistic had a value of 0.926, which is higher than 0.5, confirming the sampling adequacy. As regards Bartlett’s test of sphericity, the value of this statistic was 19,912.83, with df = 861 and *p* < 0.001, which provided evidence of a significant correlation between the items. We therefore proceeded with the exploratory factor analysis. The final items, i.e., the eigenvalues, the proportion of variance, the cumulative variance and the Cronbach’s alpha are presented in Table A2. The loadings from the nine factors extracted, had a cumulative value of 73.39% in explaining the total variance in the data.

According to the results we can affirm that the first factor explained 29.93% of the variance, the second factor 9.31%, the third factor 8.91%, the fourth factor 6.19%, the fifth factor 5.18%, the sixth factor 4.56%, the seventh factor 3.40%, the eighth factor 2.98%, and the ninth factor 2.92% of the variance. Therefore, by performing the EFA we obtained nine factors that explained the variance: factor 1—leadership condition; factor 2—self-efficacy; factor 3—colleague relationship; factor 4—job satisfaction; factor 5—the tasks; factor 6—students’ behavior; factor 7—promotion; factor 8—resources; factor 9—workload. To analyze the reliability of the nine extracted factors, we used Cronbach’s alpha, and the contribution of each element to the scale seemed to be satisfactory. According to the literature, the recommended limit value to be considered in Cronbach’s alpha is 0.60 [155]. Following the analysis, we found that the elimination of any factor did not improve the reliability of the entire scale (α = 0.919). Thus, the reliability of the proposed model in this form was established. Moreover, the reliability of each factor was calculated in the next section.

### 2.6. Reliability Analysis

In order to investigate the accuracy and the consistency of the model, we used confirmation factor analysis (CFA). In addition, for validation we used discriminant and convergent validity together with the reliability analysis (Table 2). Given the Fornell–Larcker criterion [156] for convergent validity, the average extracted variance (AVE) should be greater than 0.5. Furthermore, Hair et al. [157] considered that the AVE should be higher than 0.5 and the reliability of the composite (CR) should be above 0.7. Therefore, in order to test the reliability, we used three tests: Cronbach’s alpha (α), the average variance extracted index (AVE), and composite reliability (CR). According to the results presented in Table 2, the values of Cronbach’s alpha coefficient for every construct ranged from 0.692 to 0.995. According to the literature, there is no unanimously accepted standard that indicates what value a Cronbach’s alpha coefficient should have in order to indicate a proper fidelity. However, there are a number of benchmarks that are interpreted similarly by researchers. If the value of the Cronbach’s alpha coefficient is around 0.90 we can consider that we have excellent fidelity, if it is around 0.80 we have very good fidelity, and if the coefficient is around 0.70 we have adequate fidelity [158]. Researchers [155] consider that in the case of exploratory research, a value of 0.60 for the Cronbach’s alpha coefficients can be accepted. Therefore, the reliability of each construct that we tested was confirmed as high, since the values exceeded the recommended cut-off point of 0.6. Furthermore, in our analysis we tested the composite reliability (CR) in order to evaluate the scales with several items [159,160]. According to Kline [158], the composite reliability (CR), also called the rho-factor coefficient, is the ratio between the explained dispersion and the total dispersion. The value of this coefficient should be greater than 0.6 [160]. Thus, considering the results obtained, we can say that the factor loadings reached values in the range of 0.82 to 0.96, and these values are significantly higher than 0.60, the limit identified in the literature. Regarding the average variance extracted (AVE), all AVE values varied between 0.61 and 0.80, exceeding the cut-off point of 0.5 suggested by the literature [143,144]. Therefore, we can argue that the proposed model meets all the criteria for convergent validity.

Considering that all the analyzed items were evaluated on a 5-point Likert scale, ranging from “1 = strongly disagree” to “5 = strongly agree” and “1 = never” to “5 = always”, in our analysis we determined the mean values of the nine latent variables. Starting from these results we could identify which factor was considered by teachers as the most important and also which factor could influence job satisfaction to the greatest extent. We noted that the highest average value was efficacy (mean = 4.48), so we can say that teachers consider it very important to ask questions appropriate to the level of each student and provide alternative explanations for students in difficulty, and also that they consider it is very important to inspire students to invest in the discipline they teach.

Another factor that could influence job satisfaction is related to leadership, namely, the involvement of the school management in the activities they carry out at school. According to the results, we can say that the teachers who answered this questionnaire were of the opinion that the school management appreciated efficient teaching (mean = 4.26), that the collaboration between the school management and teachers for planning training was optimal (mean = 4.21), and that the management provided assistance whenever needed and was willing to listen to the teachers’ suggestions (mean = 4.17). In addition, according to the results, school leadership offered optimal instructional support to the teaching staff (mean = 4.14) and optimal support for professional development to the teaching staff (mean = 4.10). The teachers gave a lower value to the statement that the school leadership treated the entire teaching staff equitably. As regards the school leadership offering advice on how to improve teaching methods, the teachers considered that the advice was insufficient and must be improved. Furthermore, the workload, item “I have too much material to prepare for class” had a fairly high average value of 3.44, and hence we can say that teachers think they have too much material to prepare for class, an activity that could be considered as one that negatively influences job satisfaction. In addition, planning, developing, and organizing the teaching process (mean = 2.93) registered a fairly high average value. Thus, we can say that teachers believe that planning, developing, and organizing the teaching process is itself a process that requires the allocation of significant extra hours that do not affect the teaching process, and should be reduced. Furthermore, the reliability of each factor was also determined. The removal of any element did not seem to improve the reliability of each factor (between 0.692 and 0.995); therefore, all items were retained.

The correlation coefficient measures the strength and direction of the relationships between all the variables studied (Table 3). According to the results, there was a significantly positive correlation between job satisfaction and students’ behavior, leadership condition, resources, colleague relationships, promotion, and self-efficacy and a negative correlation between job satisfaction and tasks and workload. Regarding the correlation of job satisfaction with the other factors analyzed, we found a moderately significant correlation; the Pearson’s correlation coefficient was between −0.161 and 0.465, for n = 658 (** *p* < 0.01).

In order to examine the discriminant validity of each construct, we computed the square roots of the AVE values and compared these values with the correlation coefficients. For an acceptable discriminant validity, the square roots of the AVE values should be larger than the correlations of each construct [161]. In our case, the square roots of the AVE values of all constructs (0.80–0.90) exceeded the correlation coefficients (−0.009 to 0.577) for each construct [145]. The results in Table 4 show that the discriminant validity was acceptable.

## 3. Results

### 3.1. Structural Equations Modelling

In order to test the validity of the measurement model, we used structural equations modelling (SEM). The estimation of the model consists in obtaining some idea of the parameters that compose the reproduced matrix, so that they are similar to those in the initial matrix [162,163]. The most commonly used methods of estimating the parameters are the maximum likelihood estimation method and the generalized least squares method [164].

The identification and interpretation of the fit indexes resulting from the estimation of the model allowed us to draw some conclusions regarding the tested model. In the literature, there is much debate about the clues that should be reported in order to decide if the model is the right one. Thus, a number of authors [162,163,164] recommend the analysis of three categories of indexes: (a) goodness-of-fit measures such as chi-square, root mean squared error of approximation (RMSEA), goodness-of-fit index (GFI), and adjusted goodness-of-fit index (AGFI); (b) model comparison indexes such as the Tucker–Lewis index (TLI), normed fit index (NFI), and comparative fit index (CFI); indexes regarding the parsimony of the model such as the parsimony fit index (PFI) and parsimony normed fit index (PNFI). All of these indexes indicate a good model fit.

**Figure 2:**
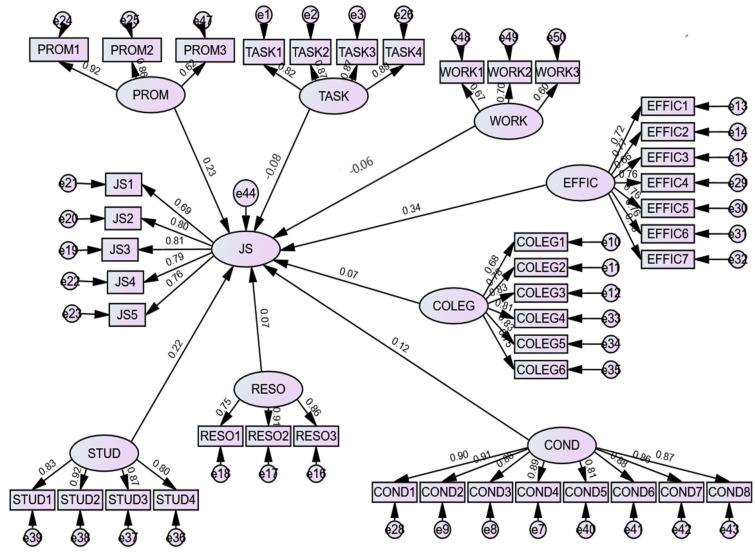
Estimates of the structural equation modelling.

Starting with the chi-square index, we can affirm that this was significant at probability level = 0.00: chi-square = 3098.065 with 852 degrees of freedom (df = 852) and chi-square/df was 3.63, which is less than 5. In order to evaluate the fit of the model, some researchers also proposed the use of the goodness-of-fit index, which takes into account the amount of variance and the predicted covariance in the reproduced matrix. A value greater than or equal to 0.90 is an acceptable value, and a GFI value of 1 indicates a perfect fit of the model [165]. Another index is the adjusted goodness-of-fit index which adjusts the GFI index to the number of degrees of freedom of the model, which is related to the number of variables in the model. A value greater than or equal to 0.80 is an acceptable value, and a value of 1 indicates a perfect fit of the model [164]. In our case these two indexes are significant: GFI = 0.909 and AGFI = 0.806. Unlike the chi-square index, RMSEA takes into account the number of estimated parameters but not the sample size. Thus, a value of RMSEA ≤ 0.05 indicates a very good model, and a value less than or equal to 0.08 shows that the model is acceptable. Because RMSEA = 0.06 we can argue that the model was acceptable. For the model comparison index, we determined the NFI (NFI = 0.916) which exceeded 0.9, indicating that the index showed an acceptable fit [148]. The literature review proposed several indexes that evaluate the parsimony of the model. A parsimonious model is the simplest or narrowest model that explains the analyzed phenomenon. In our study, the parsimonious fit index (PFI) was 0.940, while the PNFI was 0.805; both indexes are greater than or equal to 0.50 showing that the model is a suitable one [165]. In conclusion, these indexes confirmed that the proposed structural model was acceptable and suitable for the analysis and interpretation of the coefficient estimates (Figure 2).

### 3.2. Hypothesis Testing and Estimates

According to the statistical significance of the eight hypotheses proposed, we determined the standardized regression coefficients between the dependent variable and the independent variables and the significance level (*p*-value) of each coefficient, starting from the structural equations modelling output. A hypothesis is accepted when the presence of a statistically significant relationship in the predicted direction is confirmed. According to the results presented in Table 5, only three hypotheses out of the eight were accepted at a significance level of 0.01, five hypotheses at a significance level of 0.05 and seven hypotheses at a significance level of 0.10.

As Table 5 shows, all hypotheses were accepted except for H2, which was not accepted according to any of the three significance thresholds. Thus, the present findings, except for the relationship between job satisfaction and workload, are consistent with the proposed hypotheses. PROM, STUD, and EFFIC have a significant and positive impact on JB, since the coefficients (H8: β = 0.226; H5: β = 0.220; H1: β = 0.338) are significant at the 1% significance level. In case of COND (H6: β = 0.119), this also shows a positive and significant impact on JS, but at the 5% significance level, while the RESO and COLEG variables are significantly related to JB, since the coefficients are significant at the 10% significance level (H7: β = 0.066; H4: β = 0.066). Another factor that influences job satisfaction is represented by the latent variable TASK. This variable has a significant and negative impact on JB, since the coefficient, β = −0.079, is significant at the 5% level.

The strongest direct effect was found between EFFIC and JS (H1 accepted). The results suggest that when self-efficacy increases, the job satisfaction of teachers also increases. The next factor that positively influences job satisfaction is their relationships with students, followed by promotion and working conditions. Resources and relationships with colleagues also positively influence job satisfaction, but to a smaller extent. Regarding the daily tasks that teachers have to perform, the more they are in number, the lower the degree of job satisfaction of teachers. According to the results, we can say that when self-efficacy increases by 1, job satisfaction increases by 0.338, and when tasks increase by 1, job satisfaction decreases by 0.079. Therefore, hypotheses H1, H3, H4, H5, H6, H7, and H8 are statistically validated, indicating that all variables related to the relationship with colleagues, the relationship with students, and the relationship with school management significantly influence the degree of teacher satisfaction in Romania.

## 4. Discussion and Practical Implications

Job satisfaction and teacher well-being are important factors for the school environment and teaching outcomes. They are factors that keep employees from leaving their field of work altogether or merely migrating from one job to another. Companies study employee and worker migration and leaving, to assess how to create better work environments and how to stabilize the workforce in such a way as to allow dedicated and gifted employees to put their abilities and competencies into practice. They also manage the benefits of certain employees who leave the company, arguing that some employees need to leave to maximize their working potential in other fields. In other cases, leaving one’s job has positive effects, because it cancels job-related tensions.

The aim of this study was to examine the relationship between teacher job satisfaction and four main categories of determinants: self-efficacy, relational factors (colleague collaboration, student behavior, school management), work-related factors (administrative workload, teaching tasks), and working conditions, among Romanian K-12 teachers, in schools from two counties situated in North-West Romania, in order to identify various implications for teachers’ well-being. Starting from the identified relations between job satisfaction and the eight indicators, we can propose several implications that these relations may have for teacher well-being. Considering the factors that have the greatest influence on job satisfaction, namely, self-efficacy and promotion, it could be argued that teacher well-being is positively affected by a sense of professionalism, belonging, self-worth, and happiness. Teachers who have self-efficacy tend to be promoted by the school leadership, as guarantees of a high standard of education. Parents tend to choose schools according to the proven efficacy of the teaching staff. In addition, teachers who have self-efficacy tend to be encouraged to seek promotion and aided in the process through the principal’s office. Promotion results in a higher salary, social exposure, and a positive image for the school. This also increases self-worth, happiness, and an overall feeling of belonging. On the other hand, working conditions have a negative effect on job satisfaction. Working conditions refers mainly to workload, and this is a problem because it means overtime and detracts from teaching activities, which are the main endeavor of teachers. Working conditions can lower job satisfaction which, in turn, affects well-being by creating a feeling of distress, because the main objective of teaching is not accomplished. Teachers can develop higher stress levels, leading to demotivation, if workload is a constant strain.

According to the collected data, the most important factor that contributes to high levels of job satisfaction among teachers is self-efficacy in class management, subject preparations, administrative work, and emotional management. Teacher self-efficacy refers to the ability of the teacher to inspire the students to learn, both within the school environment and outside it and to adapt one’s teaching to attract student interest in a certain subject. It also aims to develop students’ abilities to think critically and to motivate low-achieving students to become involved in class activities. Self-efficacy also refers to implementing alternative teaching and learning strategies for struggling students, by formulating questions tailored to their level of development. When teachers observe the positive effects of their decisions and actions, both for the students and within the school environment, coupled with support from school management and fellow teachers, the desire to be involved increases. Being appreciated and having a strong feeling of self-worth positively influences both job satisfaction and teacher well-being. According to the results, we consider our first hypothesis (H1) to be fully confirmed. Furthermore, the study results are in agreement with previous study results (Yang et al. [84], Zakariya [55], Edinger and Edinger [79], Skaalvik and Skaalvik [80], Molero et al. [81]) showing a positive relationship between job satisfaction and self-efficacy. In addition, the results of this study showed that of all the factors analyzed, self-efficacy influences job satisfaction the most, and this is an aspect also confirmed by Zakariya [55] and Yang et al. [84]. Zakariya [55] identified self-efficacy in instruction as having the greatest impact on job satisfaction, while Yang et al. [84] found that the most substantial total effect on job satisfaction came from teachers’ self-efficacy, in both Sweden and Norway. However, the results of our study are not in agreement with the study conducted by Shaukat et al. [85], for example, who found that there was no significant correlation between a teacher’s self-efficacy, beliefs, and job satisfaction, but that a key factor was the school environment.

The study also revealed that the second factor that greatly influences job satisfaction is the possibility of professional development. The teachers that answered the questionnaire believed that teaching guarantees promotion, due to several aspects specific to the Romanian educational system. Teachers believe that if they enter the educational system, they will benefit from professional advancement. Over time, they gain expertise through various professional development programs. The study also revealed that teachers consider the teaching profession as offering a secure future, due to the level of income coupled with various incentives. Teachers seem to be aware of the possibilities for promotion and strive to achieve it, despite systemic issues. The job security offered by the education system motivates teachers to positively engage with the work environment, despite various negative aspects, and to continue being engaged. This attitude promotes higher levels of job satisfaction. Thus, we can affirm that the results support and validate the eighth hypothesis (H8) of the study. This is in line with previous research [84] which showed that professional development has a substantial effect on teachers’ job satisfaction in Sweden (0.09 for the direct effect and 0.07 for the indirect effect) and Norway (0.16 for the direct effect and 0.08 for the indirect effect), while in Finland and Denmark, only a small indirect effect was found. Toropova et al. [39] and Sims [140] also showed that professional development was positively related to job satisfaction. Toropova et al. [39], in their study, showed that teachers with a longer exposure to career and professional development seemed to have higher job satisfaction levels.

Another important factor for job satisfaction is the teacher’s relationship to the students. Within this factor, the most important aspect is the level of respect that students have for the teaching staff, followed by students’ orderly behavior, care for school property, and respect for school rules. Teachers do not seem to expect flawless behavior from students, therefore certain misbehaviors are expected, but not serious offences. Job satisfaction increases due to positive interactions with students, on the grounds that the teacher’s emotional state is balanced, avoiding attrition and burnout. Well-being is increased when students acknowledge the teacher’s efforts. The positive effect of the teacher’s relationship to the students, identified in this study, is in line with other studies that show a similar relationship [55,112]. Gil-Flores [111] found that a positive teacher–student relationship, where the teacher listens to the students, promoting the students’ well-being, played the most significant role in the prediction of teacher job satisfaction.

Leadership, in the school environment, is an important factor for job satisfaction. Our research confirmed its importance, by showing a direct and positive relation to job satisfaction. The highest score in our research was obtained in relation to leadership that appreciates efficient teaching. The image of the Romanian public school depends also on the efficiency of the teaching staff. This second-highest score was given to the collaboration between leadership and teachers for planning training. Teachers obtain points to advance in their careers by attending various training programs and going through rigorous in-class inspections. Lower scores were obtained for issues such as the leadership’s assistance to the teachers, its willingness to listen to suggestions, and the leadership’s instructional support for the teaching staff. The lowest score was obtained for the issue of the leadership’s equitable treatment of all the teaching staff. This may be explained by the fact that since the school image depends on the quality of the teaching staff, low-achieving teachers may not receive the full support of the school leadership. There is also room for extensive improvement with regard to the leadership’s ability to offer advice on how to improve teaching methods. This issue might be explained by the fact that teachers rely on their own research for the improvement of their teaching methods and styles, while the administrative issues are left in the care of the leadership. Our results are in line with other research that analyzed the relationship between the perceptions of the teachers of school-climate leadership and job satisfaction. Thus, Ainley and Carstens [120], Torres [121], Sims [122], and Liu and Werblow [123] found a positive relationship between leadership and job satisfaction, i.e., a relationship similar to the one that we identified in our study.

Job satisfaction in the teaching profession is also influenced by the school resources allocated to teachers in the teaching process. Teachers are satisfied with their jobs when they receive the necessary support for using technological material, followed by being provided with the necessary technological resources for the teaching process [126,129].

Collegial collaboration is yet another important factor that influences job satisfaction, especially with regard to collaboration with other colleagues in preparing teaching materials, followed by various consultations aimed at devising better teaching methods. Another important element is the possibility of sharing one’s expertise with fellow teachers and working in teams to implement new teaching-related strategies. In this context, teachers consider it important to be able to collaborate with fellow teachers from other classes, to ensure teaching continuity. Lastly, teachers consider feedback to be a useful tool in self-assessment and professional development. In this context, job satisfaction increases if the working environment is based on honest collegiality. Therefore, we can argue that the results of our study validate the fourth hypothesis (H4). Our results are in line with previous research, such as that of Hur, Jeon, and Buettner [103], who pointed out that collegiality contributes to increased job satisfaction and leads to positive child-centered beliefs. Furthermore, Yang et al. [84] pointed out that in Norway and Denmark, professional collaboration has the highest impact on teachers’ job satisfaction, while in Finland no significant effect was found.

The study also revealed that two of the latent variables negatively influenced job satisfaction. Thus, teachers considered that if the workload and school tasks increased, job satisfaction decreased. This is in line with Knox [87], who found that task significance was an imperative element of job satisfaction, as teachers who perform repetitive tasks are inclined to demonstrate lower job satisfaction and teachers who feel that their job is very important and believe they can make a difference in their students’ lives are likely to have higher job satisfaction. Other studies point out that teachers may have average job satisfaction levels with respect to working conditions [54,90]. However, teachers are aware of the need to perform a certain number of extra duties and that extra workload exists, but if these elements are constant, they lead to attrition, burnout, and even to teacher turnover. The latter element refers to teachers leaving the teaching profession or simply moving to other schools.

These factors influence job satisfaction and well-being in the teaching profession because they ensure a positive work environment in which teachers and students alike thrive, thus leading to higher levels of involvement from teachers, students, and parents. An efficient work environment decreases attrition, burnout, emotional exhaustion, and teacher turnover, while it increases job satisfaction, well-being, and teacher retention.

### Practical Implications

Job satisfaction is dependent on school administration and management, the teachers’ relationship with fellow professionals and with the students, and to the greatest extent on personal self-efficacy. If job satisfaction is to be increased, school managers/principals need to ensure that the teaching staff is provided with adequate professional support, access to professional development programs, sufficient teaching materials, and support for using modern technology in the teaching process. The teaching staff need to re-evaluate their relationships and increase collaboration for developing high-quality and efficient teaching materials, adequate feed-back, and support for colleagues who struggle. Teachers should also take into consideration the feedback from colleagues and other teaching staff, and parent and school-management feedback is also useful for better self-evaluation. If these elements are implemented, the school’s work environment can become more efficient and a means through which teacher attrition and burnout can be reduced or even avoided. Teacher well-being would result as a natural consequence of being valued and engaged in school decision-making processes.

In this context, teacher self-efficacy is the most important element for job satisfaction, because it refers to the personal abilities and competences that a teacher can apply in the teaching profession, in any school or educational environment, and also in other fields of expertise that may be relevant. In the school environment, self-efficacy provides the teacher with the ability to optimize and improve teaching methods, teacher–student and teacher–parent interactions, collegial collaboration, decision-making, and the school environment.

The Romanian educational system is highly bureaucratic. Various educational and administrative tasks are based on filling in papers for teaching materials, various committees, administrative decisions, requests, and so on. The system does not always allow for teachers to finish all their teaching tasks in the allocated time frame, meaning that some of the tasks need to be done outside school hours. An increase in workload lowers job satisfaction.

Overall, job satisfaction and well-being are highly dependent on how the school management/principals organize the work environment. If this is done properly, teacher self-efficacy, career promotion/career development, collegial, student, and management relationships, and the proper resources all increase job satisfaction. When teachers are valued for their efforts, fairly praised for their results, have their opinions valued and have a say in decision processes, well-being is increased. Educational/teaching tasks and administrative workload are also highly dependent on how the school management organizes the work environment. These two elements need to be carefully assessed, because if they are excessive, job satisfaction and well-being decrease.

## 5. Conclusions

This study analyzed the factors that influence job satisfaction in the teaching profession. The factors can be divided into relational factors (i.e., colleagues, students, school management), work-related factors (workload, tasks), and working conditions (work environment, attrition, burnout, self-efficacy). The results of the analysis show that the most important factor that influences job satisfaction is the teacher’s self-efficacy, which is a guarantee that the teaching process, collegial collaboration, the workload, and the tasks are handled properly and efficiently, to which one might add inspiring students with a desire to learn, leading to positive educational outcomes. The second most influential factor is represented by promotion opportunities, which are considered highly influential in job satisfaction because the educational system is organized in such a way as to give access to promotion to teachers that have tenure. This factor is followed by the respectful attitude of students, especially towards teachers, since the educational system has been plagued by negative views from within the media, thus creating higher expectations on the part of society in general, but also parents and especially students. Among the most important factors that influence job satisfaction is the support teachers receive from management, since the deciding factor in how teachers gain access to technology and school supplies depends heavily on how management operates. This aspect brings the argument to the issue of school supplies that aid teachers in delivering high-quality teaching content. The issue is just as important for online teaching, as a mix of online and face-to-face teaching is more prominent than in the past. Teaching is not dependent merely on what one teacher delivers in his or her classes, but also on how collegial cooperation works within schools. If teachers collaborate effectively, job satisfaction increases, since self-efficacy is promoted and developed at a faster pace, thus having positive effects on the educational process. With regard to the workload, it appears that this is expected to exist in higher amounts than might at first appear, since the system is still based on bureaucratic principles. However, if the workload is continuously high, and it interferes with the teaching process, this decreases job satisfaction and can lead to attrition, burnout, and even teacher turnover. Tasks are connected to workload, but they refer strictly to planning, developing, and organizing the teaching process, student evaluation, and class management, and researching new teaching methods, whereas the workload includes administrative chores. When both factors impair the teaching process, this reduces job satisfaction and well-being, by reducing the value and recognition of the teacher’s efforts and results.

### Limitations and Future Work

The limitations of this research are due to the number of variables considered. The literature on job satisfaction in the educational field includes several variables that were not included in this study. Variables such as income, background, didactic title, age, gender, and teaching tenure will be analyzed in future research projects. Along with the investigated variables that can affect job satisfaction, there is some evidence that job satisfaction may vary according to a variety of other factors, including personality, demographics, income, work experience, and teachers’ professional status [33,40,50,98,100]. Additional research is being carried out to determine the variability of the aforementioned parameters and their relationship to job satisfaction. Limitations are also due to the number of participants. Although the number is statistically relevant, the group consisted of teachers from the North-West region of Romania only. A more comprehensive study should be performed in other regions, as well as at a national level. We also consider it important to mention that cross-sectional studies imposed some predictive limitations in determining cause–effect relationships. As Carlson and Morrison [166] stated, “the main limitation of the design of the cross-sectional study is that because exposure and outcome are assessed simultaneously, there is generally no evidence of a temporal relationship between exposure and outcome”. Therefore, we consider that the inclusion of a longitudinal component at country level would be justified for an analysis of trends in teacher job satisfaction.

## Figures and Tables

**Table 1:** Respondent profile.

Characteristics	Category	Frequency	Percentage (%)
Gender	Male	590	89.75
	Female	68	10.3
Age	20–30 Years	61	9.3
	30–40 Years	167	25.4
	40–50 Years	270	41
	50–60 Years	136	20.7
	60 years or above	24	3.6
Education Level	Bachelor’s degree	322	48.9
	Master’s degree	311	47.3
	PhD degree	25	3.8
Experience	0–3 Years	39	5.9
	3–9 Years	89	13.5
	10–20 Years	208	31.6
	Over 20 years	322	48.9
Teaching Level	Kindergarten	29	4.4
	Primary	285	43.3
	Lower Secondary	174	26.4
	Upper Secondary	170	25.8
Teaching Degree	First degree	448	68.1
	Second degree	83	12.6
	No degree	127	19.3
Profession type	Educator	16	2.4
	Primary School Teacher	201	30.5
	Secondary Teacher	415	63.1
	Counseling Teacher	19	2.9
	Master Instructor	5	0.8
	Assistant Teacher	2	0.3
Residence	Urban	487	74
	Rural	171	26
Teaching Location	Urban	455	69.1
	Rural	203	30.9
Income level (RON)	Under 1500	1	0.2
	1500–2000	4	0.6
	2000–2500	30	4.6
	2500–3000	113	17.2
	Over 3000	510	77.5
TOTAL Respondents		658	100%

**Table 2:** Reliability analysis.

Latent Constructs	Items	Mean	SD	Skewness	Kurtosis	CR (above 0.6)	AVE (above 0.5)	Cronbach’s (above 0.6)
Job satisfaction (Mean = 4.47)	JS1	4.41	0.778	−1.510	2.806	0.92809	0.72095	0.903
	JS2	4.48	0.692	−1.551	3.601			
	JS3	4.59	0.661	−1.969	5.409			
	JS4	4.56	0.676	−1.816	4.402			
	JS5	4.31	0.798	−1.291	2.144			
Promotion (Mean = 3.41)	PROM1	3.28	1.114	−0.160	−0.787	0.94693	0.75655	0.837
	PROM2	3.55	1.099	−0.509	−0.454			
	PROM3	3.39	1.030	−0.245	−0.503			
Students’ behavior (Mean = 3.72)	STUD1	3.65	0.926	−0.632	−0.114	0.90271	0.79534	0.914
	STUD2	3.78	0.917	−0.678	0.023			
	STUD3	3.69	0.922	−0.572	−0.142			
	STUD4	3.77	0.912	−0.894	0.620			
Leadership condition (Mean = 4.09)	COND1	4.21	0.848	−1.289	2.085	0.96762	0.78892	0.920
	COND2	4.14	0.881	−1.085	1.070			
	COND3	4.10	0.948	−1.081	0.919			
	COND4	4.17	0.894	−1.220	1.490			
	COND5	3.79	1.039	−0.661	−0.125			
	COND6	4.17	0.888	−1.117	1.125			
	COND7	4.26	0.889	−1.420	2.102			
	COND8	3.97	1.112	−1.093	0.528			
Resources (Mean = 3.63)	RESO1	3.57	1.082	−0.519	−0.440	0.92459	0.80355	0.877
	RESO2	3.63	1.106	−0.601	−0.424			
	RESO3	3.70	1.076	−0.611	−0.335			
Collegiality (Mean = 3.60)	COLEG1	3.52	0.970	−0.117	−0.473	0.93123	0.69312	0.911
	COLEG2	3.44	1.005	−0.403	−0.276			
	COLEG3	3.93	0.832	−0.376	−0.414			
	COLEG4	3.54	1.019	−0.533	−0.142			
	COLEG5	3.76	0.960	−0.314	−0.463			
	COLEG6	3.43	1.052	−0.508	−0.091			
Workload (Mean = 2.99)	WORK1	3.44	0.970	−0.317	−0.403	0.82983	0.63924	0.692
	WORK2	2.55	0.917	0.431	0.163			
	WORK3	3.00	1.192	0.024	−0.844			
Self-efficacy (Mean = 4.48)	EFFIC1	4.54	0.564	−0.955	1.401	0.91633	0.64037	0.893
	EFFIC2	4.45	0.647	−1.097	1.929			
	EFFIC3	4.41	0.662	−0.721	−0.417			
	EFFIC4	4.36	0.665	−0.938	1.614			
	EFFIC5	4.41	0.641	−0.770	0.205			
	EFFIC6	4.58	0.563	−0.920	−0.170			
	EFFIC7	4.62	0.551	−1.120	0.562			
Tasks (Mean = 2.82)	TASK1	2.93	1.080	0.007	−0.504	0.94411	0.80858	0.921
	TASK2	2.79	1.160	0.031	−0.800			
	TASK3	2.76	1.187	0.051	−0.855			
	TASK4	2.80	1.158	0.001	−0.796			

**Table 3:** Correlation coefficient.

Coefficient									
	JS	STUD	COND	RESO	COLEG	EFFIC	TASK	PROM	WORK
JS	1								
STUD	0.426 **	1							
COND	0.401 **	0.416 **	1						
RESO	0.371 **	0.477 **	0.577 **	1					
COLEG	0.331 **	0.296 **	0.491 **	0.364 **	1				
EFFIC	0.422 **	0.270 **	0.275 **	0.234 **	0.350 **	1			
TASK	−0.161 **	−0.167 **	−0.070	−0.137 **	−0.038	−0.046	1		
PROM	0.465 **	0.453 **	0.476 **	0.435 **	0.380 **	0.268 **	−0.119 **	1	
WORK	−0.201 **	−0.229 **	−0.196 **	−0.209 **	−0.009	−0.069	0.353 **	−0.230 **	1

** Correlation is significant at the 0.01 level (2-tailed).

**Table 4:** Discriminant validity.

Discriminant Validity									
	JS	STUD	COND	RESO	COLEG	EFFIC	TASK	PROM	WORK
JS	0.85								
STUD	0.426 **	0.89							
COND	0.401 **	0.416 **	0.89						
RESO	0.371 **	0.477 **	0.577 **	0.90					
COLEG	0.331 **	0.296 **	0.491 **	0.364 **	0.83				
EFFIC	0.422 **	0.270 **	0.275 **	0.234 **	0.350 **	0.80			
TASK	−0.161 **	−0.167 **	−0.070	−0.137 **	−0.038	−0.046	0.90		
PROM	0.465 **	0.453 **	0.476 **	0.435 **	0.380 **	0.268 **	−0.119 **	0.87	
WORK	−0.201 **	−0.229 **	−0.196 **	−0.209 **	−0.009	−0.069	0.353 **	−0.230 **	0.80

** Correlation is significant at the 0.01 level (2-tailed). Square root of AVE values for every construct on the diagonal.

**Table 5:** Summary of the hypotheses’ testing results.

Hypothesis	Relationship	Standardized Regression Coefficient (β)	*p*-Value	Decision		
				0.10	0.05	0.01
H1	EFFIC -> JS	0.338	0.000	Accepted	Accepted	Accepted
H2	WORK -> JS	−0.059	0.185	Rejected	Rejected	Rejected
H3	TASK-> JS	−0.079	0.041	Accepted	Accepted	Rejected
H4	COLEG -> JS	0.066	0.092	Accepted	Rejected	Rejected
H5	STUD -> JS	0.220	0.000	Accepted	Accepted	Accepted
H6	COND -> JS	0.119	0.002	Accepted	Accepted	Rejected
H7	RESO -> JS	0.066	0.090	Accepted	Rejected	Rejected
H8	PROM-> JS	0.226	0.000	Accepted	Accepted	Accepted

## Data Availability

Data is available upon request from researchers to the corresponding author: flortan@uoradea.ro.

## Appendix A

**Table A1:** Latent variables and items.

Latent Variable	Acronym	Items (Statement)
Job satisfaction [39,57,68,71]	JS1 JS2 JS3 JS4 JS5	I am pleased being a K-12 teacher Teaching inspires me I am proud of the work I do The teaching profession encourages me to be creative and it encourages originality The teaching profession is very pleasant
Career promotion [71,152]	PROM1 PROM2 PROM3	Teaching guarantees being promoted Teaching offers the possibility of professional advancement Teaching offers a secure future
Students’ behavior [39,68]	STUD1 STUD2 STUD3 STUD4	Students’ behavior is orderly Students’ behavior is respectful towards the teaching staff Students respect school property Students respect school rules, without any serious offense
Leadership condition [39]	COND1 COND2 COND3 COND4 COND5 COND6 COND7 COND8	The collaboration between the school management and the teachers for the training planning is optimal School leadership offers optimal instructional support to the teaching staff School leadership offers optimal support for professional development to the teaching staff School leadership is willing to hear teaching staff suggestions School leadership offers advice on how to improve my teaching methods School leadership offers assistance when needed School leadership appreciates efficient teaching School leadership treats the entire teaching staff equitably
Resources [39]	RESO1 RESO2 RESO3	Teachers have adequate materials for the teaching process Teachers have adequate technological resources Teachers have adequate support for using the technological materials
Collegiality [39]	COLEG1 COLEG2 COLEG3 COLEG4 COLEG5 COLEG6	I consult with my colleagues on the proper ways to teach a certain subject I collaborate with my colleagues in preparing the teaching materials I share my expertise in teaching, with my colleagues I work in a team to implement new ideas I collaborate with professors from other classes to ensure the continuity of teaching My colleagues offer suggestions and feedback about how I teach
Workload [39,68]	WORK1 WORK2 WORK3	I have too much material to prepare for class I have too many teaching hours I have too many administrative chores
Self-Efficacy [39,68]	EFFIC1 EFFIC2 EFFIC3 EFFIC4 EFFIC5 EFFIC6 EFFIC7	I inspire the students to learn the discipline that I teach I adapt the teaching method to attract the students’ interest I develop the students’ ability to think critically I motivate the students who show little interest for the learning process I implement alternative strategies in the teaching and learning processes I offer alternative explanations to students who are in difficulty I formulate adequate questions for the level of each student
Tasks [39]	TASK1 TASK2 TASK3 TASK4	Planning, developing, and organizing the teaching process Student evaluation Research new methods of teaching Class management

**Table A2:** Factorial loads of the items.

Items	Factors								
	1	2	3	4	5	6	7	8	9
COND1	0.839								
COND2	0.851								
COND3	0.814								
COND4	0.860								
COND5	0.738								
COND6	0.862								
COND7	0.826								
COND8	0.840								
EFFIC1		0.728							
EFFIC2		0.783							
EFFIC3		0.705							
EFFIC4		0.766							
EFFIC5		0.772							
EFFIC6		0.782							
EFFIC7		0.747							
COLEG1			0.783						
COLEG2			0.839						
COLEG3			0.767						
COLEG4			0.784						
COLEG5			0.737						
COLEG6			0.774						
JS1				0.744					
JS2				0.792					
JS3				0.818					
JS4				0.797					
JS5				0.730					
TASK1					0.857				
TASK2					0.890				
TASK3					0.893				
TASK4					0.900				
STUD1						0.793			
STUD2						0.846			
STUD3						0.831			
STUD4						0.813			
PROM1							0.825		
PROM2							0.776		
PROM3							0.684		
RESO1								0.699	
RESO2								0.848	
RESO3								0.752	
WORK1									0.782
WORK2									0.677
WORK3									0.795
Eigenvalues	13.27	3.87	3.86	2.49	2.19	1.92	1.43	1.27	1.21
% of Variance	29.93%	9.31%	8.91%	6.19%	5.18%	4.56%	3.40%	2.98%	2.92%
Cumulative Variance		73.39%							
Cronbach’s alpha		0.919							
